# Aconite to Abort Colds

**Published:** 1896-04

**Authors:** 


### Aconite to Abort Colds.

The *Medical Record* strongly advocates the plan of giving aconite in the abortive treatment of colds. Small and frequently repeated doses are given, with the result that the fever is controlled, the pains in the muscles disappears and the patient put on the road to recovery. Aconite is a powerful aid in the treatment of acute bronchitis and colds in the head and chest.

BY climate, by food, by fresh air, by exercise, by rest, by bathings, by hygienic surroundings, we do much to change the soil in a sufferer from a lung complaint.

